# Effects of folk medicinal plant extract Ankaferd Blood Stopper^®^ on early bone healing

**DOI:** 10.1590/S1678-77572010000400015

**Published:** 2010

**Authors:** Sabri Cemil İŞLER, Sabit DEMIRCAN, Sırmahan ÇAKARER, Zerrin ÇEBI, Cengizhan KESKIN, Merva SOLUK, Emir YÜZBAŞIOĞLU

**Affiliations:** 1 DDS, PhD Research Assistant, Department of Oral & Maxillofacial Surgery, Faculty of Dentistry, Istanbul University, Istanbul, Turkey.; 2 DDS, Research Assistant, Department of Oral & Maxillofacial Surgery, Faculty of Dentistry, Istanbul University, Istanbul, Turkey.; 3 DDS, PhD Professor, Department of Oral & Maxillofacial Surgery, Faculty of Dentistry, Istanbul University, Istanbul, Turkey.; 4 DDS, Research Assistant, Department of Tumor Pathology & Cytology, Institute of Oncology, Istanbul University, Istanbul, Turkey.; 5 DDS, PhD, Private Practice, Istanbul, Turkey, Former Research Assistant, Department of Prosthodontics, Faculty of Dentistry, Ondokuz Mayıs University, Samsun, Turkey.

**Keywords:** Ankaferd Blood Stopper^®^ (ABS), *Thymus vulgaris*, *Glycyrrhiza glabra*, *Vitis vinifera*, *Alpinia officinarum*, *Urtica dioica*, Herbal medicines, Bone healing

## Abstract

**Objective:**

Several haemostatic agents are available for clinical use. Ankaferd Blood Stopper^®^ (ABS), a mixture of five medicinal plant extracts, has been used historically as a haemostatic agent. The aim of this *in vivo* study was to investigate the effects of ABS on early bone healing using a rat tibia defect model.

**Material and Methods:**

Sixteen male Wistar rats were randomized into two groups of 8 animals each. After deep anesthesia with ketamine, bone defects (3 mm diameter and 2 mm deep) were created in the right and left tibiae of all animals and either treated with 1 cc of ABS (Group 1) or left untreated (Group 2; control). Surgical areas were closed primarily. The animals were sacrificed on the 7th postoperative day and bone samples were collected from the tibias. The samples were examined histopathologically for infection, necrosis, fibrosis, new bone formation and foreign body reaction. The histomorphometric results were analyzed statistically by the chi square test, with the level of significance set at p<0.05.

**Results:**

Significant differences were found in both groups in terms of inflammation, necrosis and new bone formation (p=0.001, p=0.0001, p=0.001). No foreign body reaction was observed in the experimental group. ABS application decreased fibrosis in the experimental group, but there were no statistically significant differences from the control group.

**Conclusions:**

Histopathologically, it was observed that the application of ABS decreased the occurrence of inflammation and necrosis, while increasing new bone formation in early bone healing period. Further *in vitro* and *in vivo* studies are necessary for evaluating the benefits and possible adverse effects of the application of this herbal product on wound healing.

## INTRODUCTION

Bleeding can cause significant morbidity and mortality in clinical settings. Several haemostatic agents have been investigated for their role in haemostasis^6,9,11,13^.

Ankaferd Blood Stopper^®^ (ABS; Ankaferd Health Products Ltd., Istanbul, Turkey) is a traditional folk medicinal plant extract product that has been approved in the management of external hemorrhage and dental surgery bleedings in Turkey. ABS comprises a standardized mixture of the plants *Thymus vulgaris, Glycyrrhiza glabra, Vitis vinifera, Alpinia officinarum* and *Urtica dioica*. Several studies have shown that each of these plants has some effect on the endothelium, blood cells, angiogenesis, cellular proliferation, vascular dynamics and cell mediators^2,3,7,8,10,12^.Göoker, et al.^5^ (2008) investigated the haemostatic effects of ABS and reported its therapeutic potential to be used for the management of haemorrhage^5^.

Although clinical and *in vivo* studies have been reported about different haemostatic agents that are commonly used for the management of hemorrhage in clinical dentistry^6,13^, there is no evidence about the effects of ABS *in vivo* experimental models. The aim of this *in vivo* study was to investigate the effects of Ankaferd Blood Stopper^®^ on early bone healing using a rat tibia defect model.

## MATERIAL AND METHODS

### Animals and Surgery

The study was carried out in the Istanbul University, Faculty of Dentistry, Department of Oral & Maxillofacial Surgery and Institute of Oncology, Department of Tumor Pathology & Cytology. Treatment of the experimental animals was approved by the Istanbul University Animal Research and ethics Committee. experimental animals were obtained from The Laboratory of experimental Animals, DeTAM, Istanbul, Turkey.

A total of 16 twenty-week-old male Wistar rats weighing 250 to 300 g were used in this study and randomly assigned to two groups of 8 animals each. Prior to surgery, the animals were anesthetized with a 0.7 mL intramuscularly injection of a solution containing xylazine hydrochloride (Rompun^®^, Bayer, Leverkusen, Germany) and ketamine hydrochloride (Ketalar; Pfizer, New York, NY USA) at 1/0.5 proportion, 0.1 mL/100 g body weight. Surgery was performed under sterile conditions.

In the mid tibia of rats, a 5-mm long straight longitudinal skin incision was done on the front skin and, after muscle splitting (plane-by-plane muscle dissection), the periosteal membrane was stripped away to expose bone surface. A standardized ellipsoid round bone defect (5 mm in length, 1 mm in height, 1 mm in depth) was created at the anterior portion of the diaphysis of bilateral tibias, 6 mm below the knee joint using a round carbide bur (SS White, Lakewood, NJ, USA). The defect size was confirmed by a surgical stainless steel stent with the corresponding dimensions. The surgical stent was placed in the defect and confirmed visually by checking congruity to the defect wall.

Group 1 received 1 cc of Ankaferd Blood Stopper^®^ at the time of surgery, while Group 2 received no treatment and served as the control. The muscles were sutured with 4/0 catgut (Doğsan, Istanbul, Turkey), and the flaps were carefully repositioned and sutured with 3/0 black silk sutures (Doğsan, Istanbul, Turkey). Antibiotic (Sefazol, Mustafa Nevzat, Turkey) was given to the animals as an intramuscular injection intraoperatively and during 3 days postoperatively. No postoperative complications were noticed during the postsurgical course. All animals survived throughout the study period.

The rats of each group were housed into separate cages with two or three animals under climate-controlled conditions (12 h light/12 h dark; thermostatically regulated room temperature) without any restriction of mobilization. The animals of each group were sacrificed with an overdose of ketamine hydrochloride (50 mg/kg) on the 7th day after surgery, and the defects together with surrounding bone were immediately removed for histopathological analysis.

### Tissue Preparation and histopathological Examination

The specimens were fixed in 10% neutral buffered formalin overnight at 4°C, rinsed in phosphate buffered saline and decalcified in 20% formic acid solution (Merck, Darmstadt, Germany) for 10 days. The decalcified specimens were embedded in paraffin and cut into 20 semi-serial sections using a microtome (Leica Microsystemic, Germany), and routine hematoxylin and eosin (He) staining and Mallory Trichrome staining were performed. The sections were examined with light microscope under 40, 100 and 200x magnification (Nikon eclipse e600, Japan). A histomorphological review was performed by a single blinded oral pathologist to evaluate the presence of infection, necrosis, fibrosis, new bone formation, and foreign body reaction. The scores for infection, necrosis, fibrosis and new bone formation scores were determined by counting the associated cells and their ratio to the total cell count in a standardized area at 40x magnification. The ratio of cells between 0-25% was scored as *none*, 25-50% as *slight*, 5075% as *moderate*, and 75-100% as *advanced*.

### Statistical analysis

The statistical differences between the control and test groups were compared by chi square test using the GraphPad Prisma V.3 (GraphPad Software, Inc., USA) and the critical level of significance was P < 0.05.

## RESULTS

**Figure 1:**
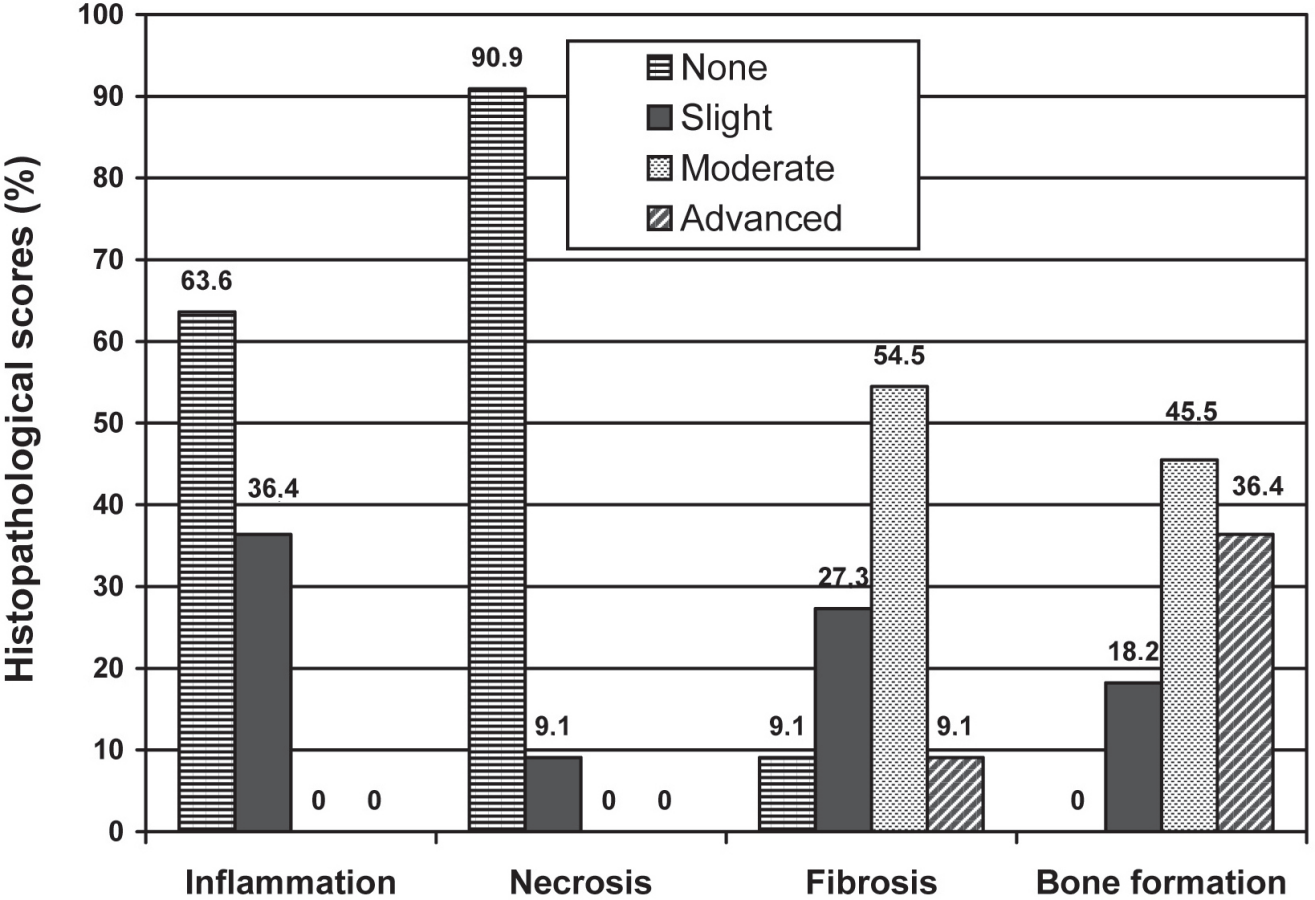
Comparison of inflammation, necrosis, fibrosis and bone formation scores in test group in percentage

**Figure 2:**
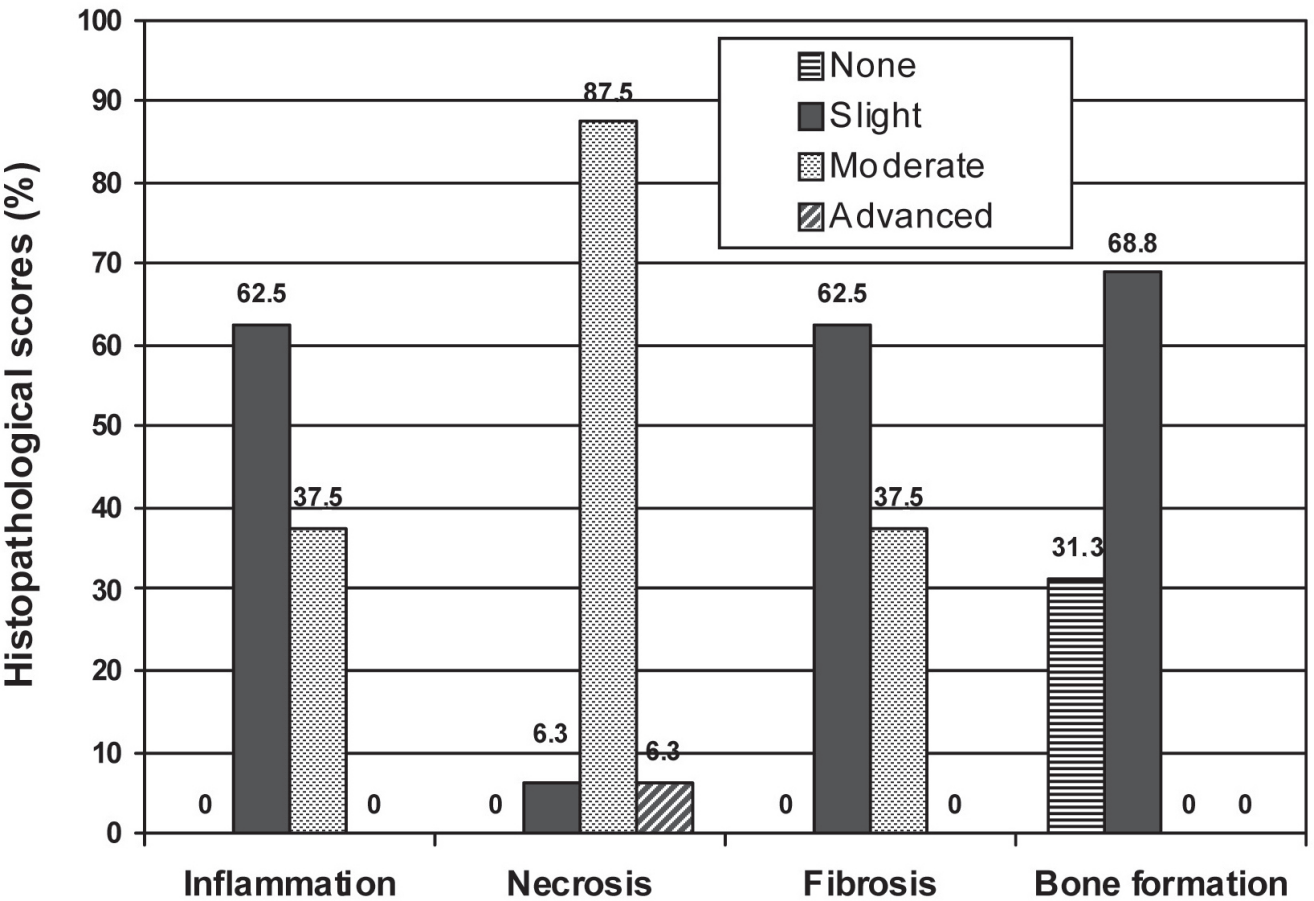
Comparison of inflammation, necrosis, fibrosis and bone formation scores in control group in percentage

The scores and percentages of inflammation, necrosis, fibrosis, and new bone formation in both groups are presented in Table 1 and illustrated in Figures 1 and 2. Comparisons between the test and control groups indicate a significant variability in the scores of inflammation, necrosis and new bone formation (p<0.001, p<0.0001, p<0.001, respectively). No foreign body reactions were seen in either of the groups.

**Table 1:** Inflammation, necrosis, fibrosis and bone formation scores in the test and control groups

	**Test group**		**Control group**	
**Inflammation**	**%**	**n**	**%**	**n**
None	63.6	7	0.0	0
Slight	36.4	4	62.5	10
Moderate	0.0	0	37.5	6
Advanced	0.0	0	0.0	0
X^2^:15.16 p=0.001				
				
	**Test group**		**Control group**	
**Necrosis**	**%**	**n**	**%**	**n**
None	90.9	10	0.0	0
Slight	9.1	1	6.3	1
Moderate	0.0	0	87.5	14
Advanced	0.0	0	6.3	1
X^2^:24.92 p=0.0001				
				
	**Test group**		**Control group**	
**Fibrosis**	**%**	**n**	**%**	**n**
None	9.1	1	0.0	0
Slight	27.3	3	62.5	10
Moderate	54.5	6	37.5	6
Advanced	9.1	1	0.0	0
X^2^:5.01 p=0.171				
				
	**Test group**		**Control group**	
**Bone formation**	**%**	**n**	**%**	**n**
None	0.0	0	31.3	5
Slight	18.2	2	68.8	11
Moderate	45.5	5	0.0	0
Advanced	36.4	4	0.0	0
X^2^:19.99 p=0.0001				

In the control group, 62.5% and 37.5% of the specimens showed slight and moderate inflammation, respectively. In the test group, 36.4% of the specimens showed slight inflammation, while 63.6% of them were free of inflammation. Statistically significant differences (p<0.001) were found between the test and control groups.

There was statistically significant difference between the groups as for the necrosis scores (p<0.0001). In the test group, 90.9% of the specimens did not show necrosis, while in the control group slight, moderate and advanced necrosis was observed in 6.3%, 87.5% and 6.3% of the specimens, respectively.

Both groups showed similar range of fibrosis scores with no statistically significant difference (p= 0.171) between them.

The results showed that bone formation scores were significantly higher in the test group than in the control group (p<0.0001). In the group treated with ABS, slight, moderate and advanced bone formation was observed in 18.2%, 45.5% and 36.4% of the specimens, respectively.

**Figure 3:**
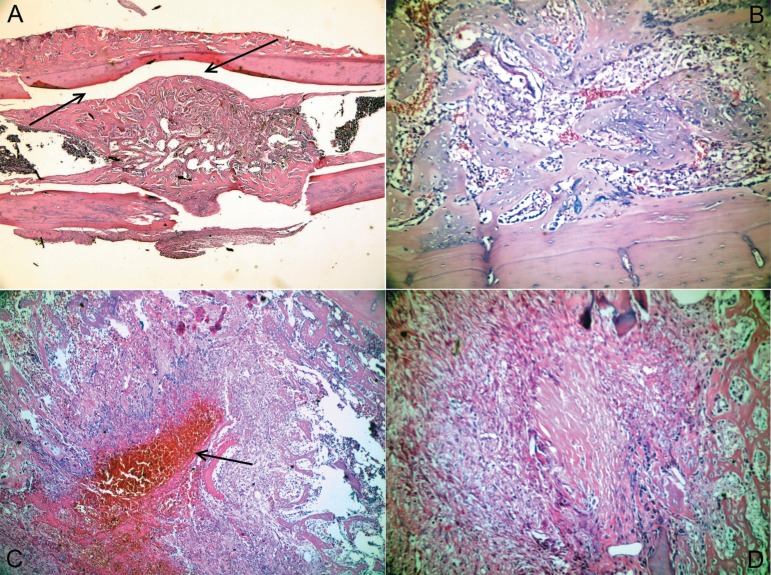
a) Non-remodeled newly formed bone tissue covering the defect area and filling the medullar space (Hematoxylin- Eosin (HE) x40). (b) Numerous new bone trabeculae in vessel-rich loose connective tissue at the medullar space (H&E x200). (c) New bone trabeculae surrounding an organizing hematoma (HE x100). (d) New bone formation areas and mild lymphocyte infiltration in an active fibrous tissue formed by mesenchymal cells (HE x200)

**Figure 4:**
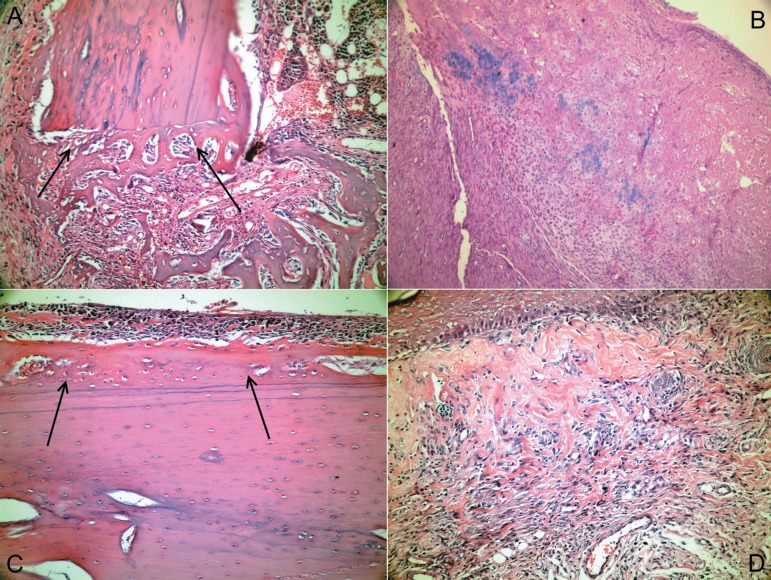
(a) New bone tissue layer separated from the defect wall by apposition lines, which is located in the loose connective tissue with mild inflammatory cell infiltration at the apex of the defect fragment (Hematoxylin-Eosin (HE) x200). (b) Areas of endochondral ossification in an active connective tissue at the defect area (HE x200). (c) Subperiostal ossification and osteoprogenitor cells that are proliferated and transforming into osteoblasts underneath the periosteum, which is traumatized near the defect area (HE x200). (d) Vessel-rich active connective tissue formed by fusiform cells underneath the surface epithelium (HE x200).

Representative histological sections of various specimens in the test group that showed decreased inflammation and necrosis, and increased new bone formation in early bone healing period are illustrated in Figures 3 and 4.

## DISCUSSION

Bleeding can cause significant morbidity and mortality in any clinical setting. Bleeding management has been studied extensively and various haemostatic agents are available for clinical use^6,13^.

ABS is a folkloric medicinal plant extract product, which has historically been used in Turkish traditional medicine as a haemostatic agent. It is a standardized mixture of the plants *T.* *vulgaris*, *G. glabra*, *V. vinifera*, *A. officinarum* and *U. dioica*, each of which has some effect on hematological and vascular parameters, and cellular proliferation^2,3,7,8,10,12^. each ingredient of this mixture has specific characteristics. *G. glabra* inhibits angiogenesis, decreases vascular endothelial growth factor production and cytokineinduced neovascularization. *G. glabra* also has antiinflammatory, anti-thrombin, antiplatelet, antioxidant, anti-atherosclerotic, and antitumor activities^10^. *T. vulgaris* has been shown to exhibit varying levels of anti-oxidant activity, which may help to prevent *in vivo* oxidative damage, such as lipid peroxidation, associated with atherosclerosis^7^. Inoculation experiments on detached leaves of *V. vinifera* exhibited enhanced resistance towards pathogens^2,3^. *V. vinifera* also has anti-atherosclerotic and antitumor effects^14,15^. *A. officinarum* inhibits nitric oxide production in lipopolysaccharide activated mouse peritoneal macrophages^8^. *U. dioica* can produce hypotensive responses through a vasorelaxation effect mediated by the release of endothelial nitric oxide and the opening of potassium channels, and through a negative inotropic action^12^.

Goker, et al.^5^ (2008) showed that the ABSinduced network formation is related to the functions of blood proteins and red blood cells. The basic mechanism of action for ABS appears to be the formation of an encapsulated protein network that provides focal points for erythrocyte aggregation. Blood cells (erythrocytes and platelets) also aggregated and participated in the network formation, with the erythrocytes forming a mass. exposure to ABS seems to provide a tissue oxygenation as well as a physiological haemostatic process without affecting any individual clotting factor. This unique mechanism of action provides ABS with an advantage over other haemostatically active plant extracts^1,4^.

The histopathological results of the present study showed that over sixty percent of the defects treated with ABS were free of inflammation, which is probably related to the antiinflammatory activity of some components of the haemostatic agent. Although the occurrence of fibrosis was statistically similar in both groups, the ABS-treated group showed lower fibrosis rate than the non-treated control group, which may be attributed to the increased speed of healing in the test group.

The defects treated with ABS also showed more intense new bone formation and less occurrence of necrosis, which may be related to the increased speed of healing and decreased inflammation which is associated with antioxidant activity of the components of the ABS.

## CONCLUSION

Within the limitations of this study, the following conclusions were drawn: 1. ABS decreased the inflammation and necrosis process; 2. ABS increased the new bone formation in early bone healing period; 3. No foreign body reaction to ABS was observed; 4. Further *in vitro* and *in vivo* studies are necessary to assess benefits and possible adverse effects of the application of Ankaferd Blood Stopper^®^ on wound healing.
